# Differential privacy protection method based on published trajectory cross-correlation constraint

**DOI:** 10.1371/journal.pone.0237158

**Published:** 2020-08-12

**Authors:** Zhaowei Hu, Jing Yang

**Affiliations:** 1 College of Computer, Jilin Normal University, Siping, P. R. China; 2 School of Automation and Software Engineering, Shanxi University, Taiyuan, P. R. China; 3 College of Computer Science and Technology, Harbin Engineering University, Harbin, P. R. China; Wuhan University, CHINA

## Abstract

Aiming to solve the problem of low data utilization and privacy protection, a personalized differential privacy protection method based on cross-correlation constraints is proposed. By protecting sensitive location points on the trajectory and their affiliated sensitive points, this method combines the sensitivity of the user's trajectory location and user privacy protection requirements and privacy budget to propose a (R,Ɛ) -extended differential privacy protection model. Using autocorrelation Laplace transform, specific Gaussian white noise is transformed into noise that is related to the user's real trajectory sequence in both time and space. Then the noise is added to the user trajectory sequence to ensure spatio-temporal correlation between the noise sequence and the user trajectory sequence. This defines the cross-correlation constraint mechanism of the published trajectory sequence. By superimposing the real trajectory sequence on the user’s noise sequence that satisfies the autocorrelation, a published trajectory sequence that satisfies the cross-correlation constraint condition is established to provide strong privacy guarantees against adversaries. Finally, the feasibility, effectiveness and rationality of the algorithm are verified by simulation experiments, and the proposed method is compared with recent studies in the same field on basis of merits and weakness and so on.

## 1 Introduction

The rapid development of mobile social networks and mobile positioning devices has added convenience to people's lives, but a large amount of personal location and trajectory data have been collected by third-party organizations. Systematic analysis and mining of these trajectory data can provide much useful information, and can facilitate the leakage of personal privacy information. With the advent of the era of big data, the intensive integration of massive user trajectory data with multi-source data makes it difficult to provide effective privacy protection of sensitive information with traditional privacy protection methods designed for small amounts of data. It is important to ensure that the sensitive information of users is not leaked as the availability of trajectory data is improved in privacy protection strategies.

Existing trajectory privacy protection methods are mainly based on the trajectory k-anonymity or differential privacy protection method. These methods generalize or confuse the entire trajectory to make an attacker unable to distinguish a real user trajectory from k trajectories, or make the real user trajectory unable to be identified due to the addition of noise [1–11]. However, with the advent of the era of big data, a large number of data types, low data density and fast collection speed, the existing trajectory privacy protection methods cannot fully meet the increased privacy protection requirements.

On one hand, the current trajectory privacy protection mechanism performs the same degree of anonymous processing for the entire trajectory, and ignores the potential impact of the user's context on trajectory privacy protection, which reduces the availability of trajectory data and makes the published trajectory data useless. On the other hand, the differential privacy-based trajectory protection method mainly adds scrambling noise so that an attacker cannot identify the user’s real information from the published trajectory data. However, the addition of Laplacian noise or exponential noise is basically independent and irrelevant, making this kind of noise easily removed by the attacker through application of certain filtering methods, thus limiting the overall privacy protection effect and data utilization.

In order to resolve problem of low privacy protection and poor data availability, a personalized differential privacy protection method based on cross-correlation constraints is proposed to achieve personalization of privacy protection and noise disturbance levels, which is better than the current methods.

In the first place, to solve the problem of low published trajectory data utilization which is due to application of the same intensity of privacy protection processing on the user trajectory, the (R,Ɛ)-Differential privacy protection model is proposed. While protecting the sensitive location points on the user trajectory, this model also protects the affiliated points located near the sensitive points. This approach assigns different privacy level values according to the difference of location point sensitivity. Additionally, this model sets different differential privacy budgets and provides different degrees of privacy protection, which can improve the availability of published trajectory data.

Furthermore, to solve the problem of low degree of privacy protection caused by poor correlation of the added noise signal, the original trajectory and the published trajectory, cross-correlation constraints of the published trajectory sequence are proposed based on the literature [1]. Additionally, the added noise sequence and the original trajectory sequence are auto-correlated in this method, and the cross-correlation constraints are used to ensure the consistency and indistinguishability of the published trajectory sequence, original trajectory sequence and the noise sequence in logic and spatio-temporal resolution, which can improve privacy guarantee. The main contributions of this work are as follows:

Firstly, in order to provide different degrees of privacy protection according to the context of the mobile user's trajectory and the sensitivity of the location points, the (R, Ɛ)-differential privacy protection model is proposed. It protects the privacy information of the entire trajectory by protecting the sensitive location points and affiliated points, which are directly connected to the sensitive location points. Privacy level values are assigned according to the differences of location point sensitivity. Additionally, differential privacy budget parameters are set and different intensities of disturbing noise are added, which further improves the usability of the published trajectory data.

Secondly, it proposes a differential privacy trajectory protection method which is based on cross-correlation constraints of published trajectory sequence. The method introduces the cross-correlation function of the trajectory sequence, and it gives the cross-correlation constraints that must be satisfied by the published trajectory sequence. This approach closely integrates cross-correlation and autocorrelation and limits the cross-correlation of the published trajectory sequence. In this way, the method ensures the consistency and indistinguishability of the published trajectory sequence, the real trajectory sequence and the noise sequence in logic and spatio-temporal correlation, which enhances the privacy protection effect.

Thirdly, the feasibility and effectiveness of the proposed method are demonstrated by comparing with the other two privacy protection methods for analysis of a real data set. The results show that the proposed method can provide efficient privacy protection and good published trajectory data availability, and the proposed method is compared with recent studies in the same field on basis of merits and weakness and so on.

The remainder of this paper is organized as follows: Section 2 discusses related work, and section 3 makes a statement about the research question and presents some basic concepts. Section 4 proposes a privacy protection mechanism based on location-affiliated sensitive points, and it proposes a privacy protection mechanism based on the published trajectory sequence cross-correlation constraints. In section 5, experiment discussion and analysis are shown and proposed method is compared with recent studies. Section 6 concludes the paper and discusses the future direction.

## 2 Related work

With the development of location-based services and data mining technologies, methods of data protection are a significant focus in the field of information security. Most current trajectory privacy protection methods are based on suppression, disturbing, generalization and encryption. In 2002, Latanya Sweeney proposed a formal privacy protection model named k-anonymity to protect the privacy of published data, which formed the basis of many privacy protection methods in the later period [2]. The k-anonymity privacy protection model is the most widely used generalization-based privacy protection model. In this approach, the user's real trajectory must be anonymized with k-1 other trajectories, so that the probability of an attacker identifying the user's real trajectory is no more than 1/k.

In 2006, Dwork et al. proposed a differential privacy protection method to protect the leaking of private information by adding random disturbed noise to the data set [3,4]. This approach was based on a rigorous mathematical model, and has been deeply researched and widely used as the basis of many privacy protection methods. Xiao Zhe et al. proposed a method to protect trajectory privacy by using query logic to separate the storage mechanism [5], with non-cluster location tuple for client trajectory reconstruction and server-side privacy protection to solve the personal variable privacy problem. Marco Fiore surveyed the privacy protection issue of the trajectory micro-database and proposed a solution to protect the database from attacks [6]. Wang Hao et al. proposed a correlation differential privacy noise addition method based on the sequence autocorrelation function [1], which ensured the indistinguishability of anonymous trajectories by adding noise that was autocorrelated with the user's real trajectory sequence.

Yan Dai et al. proposed a personalized trajectory privacy protection method [7] that marked the semantic properties of all sample points on the trajectory and built a corresponding classification tree. Sensitive stop points were then extracted and different strategies were used to select the appropriate user interest points for different types of sensitive stop points. Zhu Weijun et al. proposed a differential privacy trajectory protection method based on statistical methods in a vehicle networking environment [8]. The method calculated the sensitivity of location points by analyzing the vehicle trajectory characteristics to add Laplacian noise based on the sensitivity. Konstantinos considered the meaning of differential privacy when the indistinguishability of two adjacent databases depends on an arbitrary notion of distance, and gave intuitive describes of the privacy threat according to Bayesian adversaries [9]. Jin Kaizhong et al. proposed a differential trajectory-privacy protection method based on an adaptive method [10]. In this approach, sequence truncation was used to decrease the global sensitivity of the trajectory sequence, which can reduce data processing errors. The frequent pattern mining algorithm of prefix sequence lattice was used to decompress the scale of frequent trajectories and reduce generated redundant sequences, which avoided the repeated assignment of Ɛ.

Abiodun encrypted the information in the social network, transformed the message into an unreadable form through encryption technology, and actively prevented eavesdropping attacks to achieve the protection of private information [11].Liu Xiaoqian et al. proposed a differential privacy protection method based on clustering anonymization [12]. The method divided the data set into several equivalence classes according to its distribution density, added Laplacian noise to each data equivalence class, and then generalized the sensitive properties of the query function to achieve privacy protection. Tian Ye et al. proposed a semantic tree-based algorithm [13], which constructed the fuzzy region from the stealth algorithm as a semantic region tree. Weight values were then assigned to regions based on their popularity to determine the similarity of spatio-temporal relationships. Sun Kui et al. proposed an enhanced differential privacy data algorithm to improve the availability of anonymous data [14]. The method generalized real data, used an exponential mechanism to select an optimization scheme in the specialization iteration process, established the equivalence class of the decision tree, and then added noise disturbance to create the published data.

Zhang Lin et al. proposed a method to protect the privacy of location in big data published information based on a differential privacy protection model [15]. The method used spatial decomposition technology to decompose two-dimensional positions into data sets and then used a quad-tree to iteratively process to allocate and adjust privacy budgets, which improved data availability while protecting private information. Hector Page gave a detailed introduction to the application of differential privacy in statistics of government departments, and systematically explained the technical advantages and application fields of differential privacy, which provided a basis for government departments to use differential privacy methods for census of population data [16]. Li Hongcheng et al. proposed a k-means algorithm based on differential privacy [17] in order to solve the privacy leakage caused by the malicious analysis of private information by attackers in a distributed environment.

**Fig 1:**
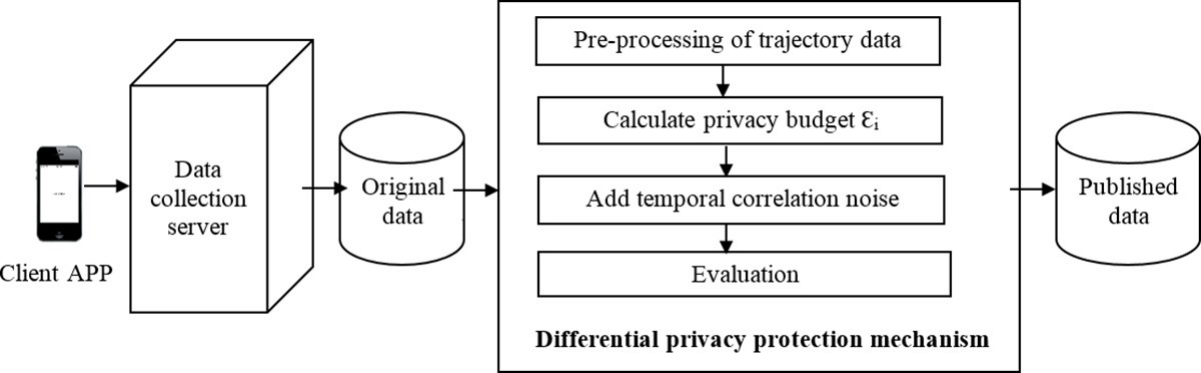
Differential privacy protection model structure.

The difference between this work and previous research is as follows. The first place, to improve data availability, the entire trajectory is protected by protecting sensitive location points of the trajectory and affiliated sensitive points. According to the difference of sensitivity, different privacy level values are set, different privacy budget values are assigned, and noise of different intensities is added, which improves data utilization while protecting private information. The second place, in order to enhance the privacy protection effect, the correlation constraint of the published trajectory sequence, original trajectory sequence and the noise sequence is set, and this constraint considers their autocorrelation and cross-correlation. Autocorrelation is used to ensure the consistency of the added noise sequence and the original trajectory sequence in timing series. Cross-correlation guarantees the consistency of the published trajectory sequence, the original trajectory sequence and the noise sequence in logic, space and time, to further improve the privacy protection effect. The differential privacy protection model structure as shown in Fig 1, it can be applied to privacy protection in the fields of Internet big data analysis, mobile social networks, traffic management, route planning, urban planning, crime detection, medical big data, bank financial securities and government census statistics.

In this model, firstly, user’s trajectory data is collected by the data collection server, then trajectory data is processed and divided into sensitive location and non-sensitive location, and to construct an undirected graph of the trajectory. Secondly, According to the differential privacy protection model condition, the privacy budget Ɛ_i_ is calculated corresponding to each location point. Thirdly, according to the defined time interval and privacy budget, it is to add Laplacian noise with correlation and varying scrambling intensity to each location point to be protected. Fourth, when the trajectory data is evaluated to meet privacy protection requirements, then obtained cross-correlation trajectory sequence is published.

## 3 Problem statement

### 3.1 Problem characterization

Current trajectory privacy protection mechanisms perform the same degree of anonymous processing on the entire trajectory, but ignore the influence of the context and location sensitivity of the user trajectory on the privacy protection effect. Applying the same degree of anonymity for all trajectories does not meet the privacy protection requirements of users. Users may have lower privacy protection requirements for some places, and if high-intensity anonymous processing is used in these places, the utilization of data will be reduced and the published trajectory data will be useless. In other places, there may be higher privacy protection requirements, and use of the same privacy protection method will alter the privacy protection effect and make it insufficient for effective privacy protection. At the same time, adding the correlation of disturbing noise to the user's real trajectory and the correlation of published the trajectory also exert a great influence on the privacy protection effect.

The results of this work provide insight into how to scientifically and quantitatively set the privacy budget based on the difference characteristics of location point sensitivity, to add disturbing noise of different intensities. This approach can ensure the consistency of the published trajectory sequence with the original trajectory sequence and the noise sequence in logic, space and time, in order to meet the personalized and differentiated privacy protection requirements of users.

**Fig 2:**
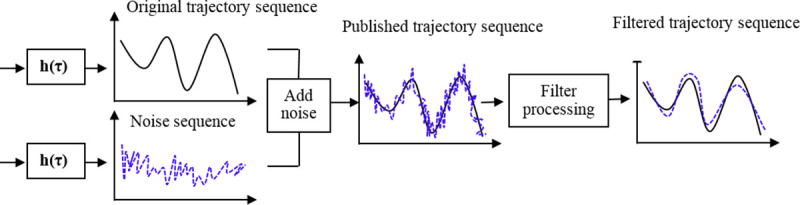
The impact of time series correlation on privacy protection.

In addition, the correlation of time series will increase the global sensitivity of differential privacy, many existing methods try to reduce the global sensitivity of the correlation time series to protect privacy. In fact, the correlation between the added noise sequence and the original trajectory sequence is very different, and the correlation between the published trajectory sequence and them is even more important. This correlation of sequences can be fully exploited by attackers to increase the probability of successful attack, as shown in Fig 2, the more background knowledge of sequence correlation is mastered by an attacker, the more noise will be eliminated, and the attacker can identify the target user with a high probability. Differential privacy adds noise to statistics, consistency and negative counts are the important problems of privacy protection, its research on autocorrelation consistency constraints is to ensure data consistency and private guarantee.

### 3.2 Basic concepts

In 2006, Dwork et al. proposed a differential privacy protection model based on data that had been distorted using strong privacy protection. The proposed model had a strict mathematical model structure and showed privacy information leakage both qualitatively and quantitatively. In this work, a personalized differential privacy protection method based on cross-correlation constraints is proposed to achieve personal privacy protection and maximize noise disturbance levels.

**Definition 1 ℇ -differential privacy.** A randomized function F: D → D is ℇ -differentially private if for all neighbouring proiles x, x ′∈D and for all t∈D [18].

$$\frac{\mathrm{Pr}[f(x)=t]}{\mathrm{Pr}[f(x')=t]}\leq \exp^{\varepsilon }$$ (1)

where the probability is computed over all the coin tosses of F.

#### Definition 2 Laplace mechanism

In a given dataset *D*, function *R*: D→R^D^, and the global sensitivity *Δf*, if R satisfies the following conditions, R is said to satisfy ℇ-differential privacy protection. The calculated noise value complies with the actual value of Laplace distribution, which is noise∼Lap(*Δf* /Ɛ) [19].

$$R=R(D)+Laplace(\frac{\Delta f}{\varepsilon })$$ (2)

#### Definition 3 sensitive location set

A sensitive location is a location that a user considers to be private and does not want to be revealed. This may be a location where a mobile user sometimes visits for a specific purpose, which the user may not want other to know about, such as drug rehabilitation centers and prisons. A set of sensitive location points is called a sensitive location set. According to the user's privacy protection requirements, when the user passes through a location point in the sensitive location set, the location point needs to be protected. The set of sensitive locations is generally pre-set by the user or the system, and it is specifically expressed as: PL = {PL_1_,PL_2_,…,PL_n_}.

#### Definition 4 non-sensitive location set

At a non-sensitive location, when a user is at the location, the user's location information can be completely disclosed without any privacy protection, such as shopping malls and parks. A set of non-sensitive location points is called a non-sensitive location set, and is specifically expressed as:NPL = {NPL_1_, NPL_2_,…, NPL_n_}.

#### Definition 5 logical location set

Logical location points are locations that are rarely accessed in daily life, but do exist. These locations may be very different from the usual or daily locations of a user. The set of these points is called a logical location set, and is specifically expressed as:LL = {LL_1_,LL_2_,…,LL_n_}.

#### Definition 6 undirected trajectory graph

The trajectory graph TG = (V, E, W) is an undirected graph composed of vertex V, edge E, and weight W, where *v*_*i*_(v_i_∈V) represents the area in the map, *i* (i = 1,2,…,n) is the number of the area in the map. If any two areas v_i_ and v_j_ are reachable, an edge *e*_*ij*_ (*e*_*ij*_∈E) is established from the vertex v_i_ to v_j_ (v_j_ to v_i_) in the TG. The weight of the edge *e*_*ij*_ is expressed as *w*_*ij*_(w_ij_∈W), which represents the Manhattan distance between the particles centers of regions v_i_ and v_j_.

**Fig 3:**
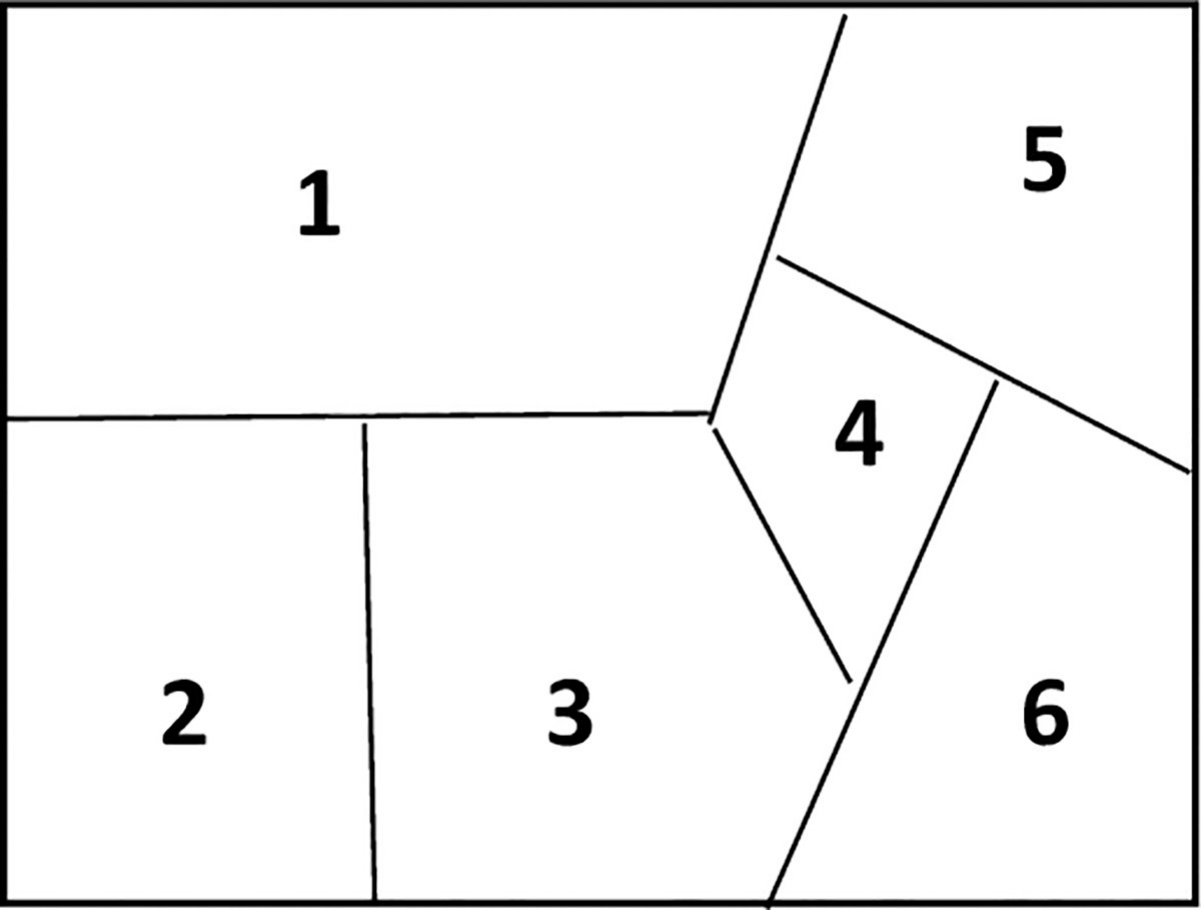
Semantic map.

When constructing the undirected trajectory graph, the entire regional map is meshed and constructed into a semantic map. The semantic map is divided into different map areas, each area is numbered, and the privacy level value is set and calculated, as shown in Fig 3. Finally, the semantic map topology of the user movement is constructed. The entire map area included three sets of location points: sensitive location set, non-sensitive location set, and logical location set, expressed as: L_Map_ = {PL,NPL,LL}.

**Fig 4:**
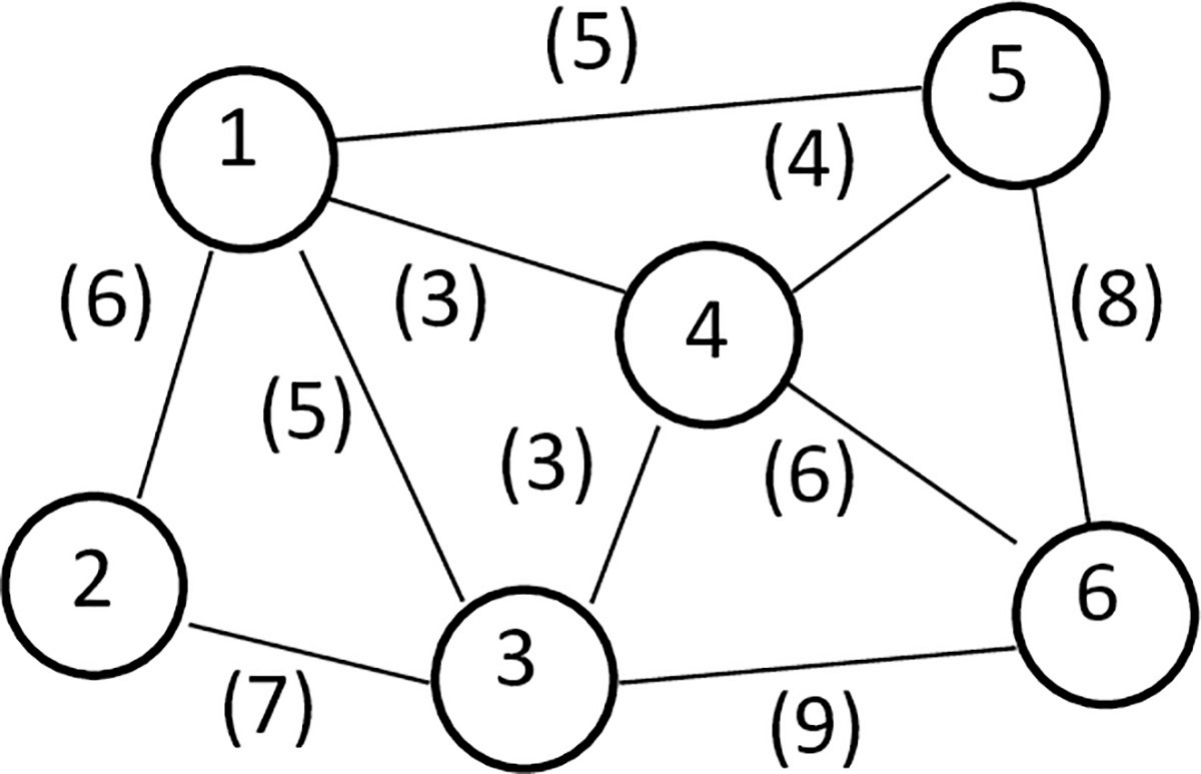
Undirected trajectory graph.

Fig 4 shows a formed trajectory undirected graph TG of Semantic map, where v_1_ and v_3_ represent two position areas numbered 1 and 3. The line between these areas is represented as edge e_13_, and this line indicates the two areas could be connected. The value 5 in parentheses on edge e_13_ indicates that the Manhattan distance between the centroids of this region is 5.

#### Definition 7 privacy protection level value

The privacy protection level value refers to the numerical representation of the sensitivity level corresponding to each sensitive location point. The value of the privacy level value is PV∊ [0,1]. The larger the value of the privacy level, the more sensitive the location point. Assuming Set_PV_ = {pv_1_, pv_2_,…, pv_i_,…, pv_n_} is a set of privacy protection level values, and pv_1_> pv_2_>…> pv_i_>…> pv_n_, then the privacy protection level value pv_i_ is calculated as:

$$pv_{i}=\left\{ \begin{array}{l} f(pv_{1})=1\\ f(pv_{i})=\frac{n-i}{n-1}\ldots .1<i<1\\ f(pv_{n})=0 \end{array}\right.$$ (3)

The privacy level value of a location point on the trajectory refers to the privacy level value of the map area in which the location point is located. In the trajectory undirected graph, if any vertex v_i_ is a sensitive position point, the privacy level value at the position is greater than zero, such as PV_i_ = 0.4. If it is a non-sensitive location point, the privacy level is 0. In the actual trajectory privacy protection, since the user's trajectory has a certain space-time correlation, the non-sensitive location points are directly connected to sensitive location points and also show certain sensitivity. If these points are not protected, sensitive information may be disclosed. Therefore, it is necessary to protect the location points that are associated with sensitive location points to further improve the privacy protection level. In this work, the location points which are associated with sensitive location points are referred to as affiliated sensitive points.

#### Definition 8 affiliated sensitive point

A point of the mobile user's trajectory that is directly connected to a sensitive location point is called an affiliated sensitive point. Affiliated sensitive points are significantly correlated with sensitive points on the trajectory, which have a great influence on the privacy protection effect of the user's trajectory. This proposed method protects sensitive points on the trajectory as well as these affiliated sensitive points. Logically, the sensitivity of an affiliated sensitive point is related to the distance from a sensitive point. The closer this distance is, the higher the sensitivity, and the larger the corresponding privacy level value. Conversely, the farther away the points, the lower the sensitivity, and the smaller the corresponding privacy level value. The privacy level value of the affiliated sensitive point is calculated as:

$$v_{i}.pv=pv\cdot \frac{\frac{1}{dv_{i}}}{\sum_{i=1}^{m}\frac{1}{dv_{i}}}$$ (4)

Where p_v_ represents the privacy level value of the sensitive location point *v*, v_i_(v_i_∊N) denotes any affiliated sensitive point directly connected to *v*, *d*_*vi*_ represents the Manhattan distance between *v*_*i*_ and *v*, *m* represents the number of elements in the affiliated sensitive point set N, which is the same as the degree of the vertex *v* in the TG, *v*_*i*_.*pv* represents the privacy level value of affiliated sensitive point *v*_*i*_.

When the degree of the sensitive node *v* in the undirected graph TG is 1, the privacy level of its affiliated sensitive point is the same as the privacy level of the original point. When the degree of the sensitive node *v* is greater than 1, the smaller the distance between the affiliated sensitive point and the sensitive location point and the larger the obtained privacy level value. If an affiliated sensitive point *v*_*i*_ is also a sensitive location point, the privacy level value of *v*_*i*_ is the higher of the assigned privacy level value and the privacy level value of that point.

For example, in Fig 4, the initial privacy level of sensitive vertex v_4_ is 0.7, the degree of v_4_ is 4, and the affiliated sensitive points connected to it are {v_1_,v_3_,v_5_,v_6_}. The Manhattan distance of these nodes and v_6_ is 3, 3, 4, 6, respectively, so the privacy level values assigned to these nodes are:

$$v_{1}.p_{V}=0.7\times \frac{\frac{1}{3}}{\left(\frac{1}{3}+\frac{1}{3}+\frac{1}{4}+\frac{1}{6}\right)}=0.7\times \frac{4}{13}=0.22$$

$$v_{3}.p_{V}=0.7\times \frac{\frac{1}{3}}{\left(\frac{1}{3}+\frac{1}{3}+\frac{1}{4}+\frac{1}{6}\right)}=0.7\times \frac{4}{13}=0.22$$

$$v_{5}.p_{V}=0.7\times \frac{\frac{1}{4}}{\left(\frac{1}{3}+\frac{1}{3}+\frac{1}{4}+\frac{1}{6}\right)}=0.7\times \frac{3}{13}=0.16$$

$$v_{6}.p_{V}=0.7\times \frac{\frac{1}{6}}{\left(\frac{1}{3}+\frac{1}{3}+\frac{1}{4}+\frac{1}{6}\right)}=0.7\times \frac{7}{30}=0.11$$

The distances of nodes v_1_, v_3_, with v_4_ are the same, so the obtained privacy level values are also the same. The distance between nodes v_4_ and v_6_ is the farthest, and the obtained privacy level value is the smallest. The node v_5_ itself is a sensitive node with an initial privacy level of 0.4, which is larger than the assigned privacy level value, so its final privacy level is 0.4.

## 4 Proposed methodology

### 4.1 Extended differential privacy protection model

This paper proposes a point extension method according to the characteristics of sensitive location points and affiliated sensitive points on user trajectories, where points are protected rather than globalizing the entire trajectory for the purposes of safeguarding privacy. The proposed privacy protection model is also based on the differential privacy protection method, where various privacy levels are determined according to the sensitivity of location points. Location point sensitivity values, privacy levels and differential privacy budget parameters are combined with various noise characteristics and degrees of sensitivity to achieve efficient and effective privacy protection.

#### Definition 9: (R, Ɛ)-differential privacy protection model

If the privacy level value *pv* of a location point and its corresponding privacy budget parameter *Ɛ* satisfy the condition *pv*×*Ɛ* = *R*, then the differential privacy protection model necessitates (R, Ɛ)-differential privacy protection.

In this case, R is the privacy model parameter which is set by the user or the system, R∊(0,1]. When R is fixed, the privacy level value in the location point is large and the privacy protection budget is small, more noise may be added and the privacy protection level is stronger. When the privacy protection level value pv = 1, the differential privacy budget parameter is a fixed constant, the privacy budget does not change with the user's requirements or the sensitivity of the location. In this case, the (R, Ɛ)-differential privacy protection model is, equivalent to the traditional Ɛ-differential privacy protection model. The differential privacy budget parameters can be changed by introducing the privacy model parameter R. This also alters the privacy protection level of the location points on the trajectory, and allows differentially disturbed noise to be dynamically added to alter the differential privacy protection effects.

Location sensitivity, user requirements and privacy budget characteristics are combined in the proposed (R, Ɛ)-differential privacy protection model. Differential privacy parameters can be calculated based on the user's location and privacy requirements, and in order to add differential Laplacian noise as necessary. The sensitivity threshold parameter Δ is adjusted to ensure the privacy level value of the sensitive point satisfies pv≥Δ for anonymous processing, which can further enhance the efficiency of privacy protection (Algorithm 1).

In addition, after obtaining the corresponding differential privacy budget parameters based on the location sensitivity, privacy protection level and differential privacy model parameter, the privacy level of location and privacy budget are fixed. According to the combined nature of differential privacy protection elements, if each location point *loc*_*i*_ on the trajectory T meets Ɛ_i_-differential privacy protection, the entire trajectory also satisfies Ɛ_i_-differential privacy protection.

**Algorithm 1.** Calculate privacy level values and differential privacy budget parameters

**Input:** trajectory graph TG = (V, E, W), trajectory map area set L_Map_ = {PL, NPL, LL}, initial privacy level value PV, differential privacy model parameter R, privacy protection parameter threshold Δ

**Output:** final sensitive location set PL, privacy level value pv, privacy budget Ɛ_i_

1 PV = P_v0_

2 V = PL

3 while (V≠Ф) then

4 for (∀v_i_∈PL)

5 Look for a affiliated sensitive point set which is directly connected to v_i_ V_i_.nei = {g_1_,g_2_,…,g_m_}

6 if v_i_. p_v_≥Δ, then

7 Calculating the Manhattan distance between g_i_ and v_i_ in V_i_.nei, and building the distance set DV = {dv_1_, dv_2_,…, dv_m_}

8 Calculating the corresponding privacy level value for each node g_i_ in v_i_.nei //formula (4)

9 if g_i_∈pL^initial^, then

10 g_i_. p_v_ = max(g_i_. p_v_, g_i_. p_v initial_)

11 P_v_ = P_v_∪ {g_i_. p_v_}

12 PL = PL∪ {g_i_}

13 ℇ_i_ = R/p_vi_

14 ℇ = ℇ∪ {ℇ_i_} 

15 end if

16 end for

17 end while

18 Return PL,PV, ℇ

In Algorithm 1, when each vertex is connected to other vertices in an undirected graph, the algorithm loops the greatest possible quantity of iterations with maximum time complexity of O(n(n-1)). In Step 6, when the privacy level value of the location point exceeds the threshold Δ, a privacy level value is assigned to the affiliated sensitive point. This enhances the execution efficiency of the privacy protection method.

After obtaining the privacy budget parameter Ɛ_i_ which corresponds to all location points of the trajectory, different levels of disturbing noise can be added at various locations to protect private information under the the (R,Ɛ)-differential privacy protection model condition. However, there is a certain spaio-temporal correlation of the location points on the user trajectory. If the correlation of the published trajectory sequence is inconsistent with the original trajectory and noise sequences, the privacy protection effect degrades to a certain extent (potentially to the point of leaking private information). In this study, cross-correlation constraints of the published trajectory sequence are established based on the (R,Ɛ)-differential privacy protection model. This ensures that the noise sequence is related to the original trajectory sequence, which further ensures a logical and spatio-temporal correlation with the published trajectories for effective privacy protection.

### 4.2 Cross-correlation constraint for published trajectory sequences

When operating the proposed method, the added noise is generally Laplacian noise or Exponential. The proposed privacy protection mechanism is based on previously published trajectory sequence cross-correlation constraints. Gaussian white noise is converted into Laplacian noise by setting the power spectral density and shock impulse response of the filter, then adding them to the user's real trajectory sequence. This determines the constraint mechanism that need to be satisfied with published trajectory sequence through autocorrelation functions and cross-correlation functions for efficient privacy protection.

#### Definition 10: Autocorrelation function

The autocorrelation function is an impact function which describes the dependence of a random function or signal sequence in two consecutive time points *t*_*1*_ and *t*_*2*_. It also reflects the correlation between random function or signal in time *t* and (*t+τ*). It is expressed as follows:

$$R_{{(t}_{1}{,t}_{2})}^{f}{=E[f(t}_{1}{),f(t}_{2})]$$ (5)

For continuous functions, it is expressed as:

$$R_{{(t}_{1}{,t}_{2})}^{f}=f(\tau )*f^{*}(‐\tau )=\int_{-\infty }^{+\infty }f(t+\tau )f^{*}(t)dt=\int_{-\infty }^{+\infty }f(t)f^{*}(t-\tau )dt$$ (6)

**Definition 11: Cross-correlation function.** The cross-correlation function describes the degree of correlation between two random functions or signal sequences in two consecutive time points t_1_ and t_2_. It also describes the correlation of random functions or signals f_1_(x) and f_2_(x) in any two consecutive time points t and (t+τ). It is expressed as:

$$R_{{(t}_{1}{,t}_{2})}^{{(f}_{1}{,f}_{2})}{=E[f}_{1}{(t}_{1}{),f}_{2}{(t}_{2})]$$ (7)

For continuous functions, it is expressed as:

$$R_{{(t}_{1}{,t}_{2})}^{{(f}_{1}{,f}_{2})}=\int_{-\infty }^{+\infty }f_{1}^{*}f_{2}(t+\tau )dt$$ (8)

The user trajectory sequence is spatio-temporal related and the added Laplacian noise elements are independent of each other, so the attacker can filter out these independent pieces of noise based on background knowledge to identify real private information. If there is a temporal correlation of the published trajectory sequence with the user's real trajectory sequence and the added Laplacian noise sequence, i.e., relation in terms of spatio-temporal and contexual information, then the attacker cannot identify the real user trajectory from the published trajectory sequence and the user's privacy is protected.

#### Definition 12: Timing correlation

If the autocorrelation function RX'(τ) of the added noise sequence is the same as the autocorrelation function RX(τ) of the user's real trajectory, then RX'(τ) = RX(τ). In this case, the noise sequence and the user's real trajectory sequence are time-series correlations. Adding the noise sequence to the user's real trajectory prevents the attacker from identifying the user's real trajectory information from the published trajectory sequence logically.

Timing correlation ensures that the added noise is logically consistent with the original trajectory sequence. A noise sequence related to the user's real trajectory timing is added which does not increase the additional noise intensity but does ensure the same privacy protection as the original differential privacy. Therefore, there is no impact on the availability of trajectory data.

The temporal consistency of mobile user's trajectory sequence can be measured according to the characteristics of the autocorrelation function. The consistency of added noise sequences in different time points can be measured by the autocorrelation function of the noise sequence. The independent signal is input and processed by the filter to make it non-independent according to the signal system characteristics. The new sequence formed by the two correlation sequences after filter processing does not necessarily complete the correlation in the spatio-temporal domain. In this paper, the cross-correlation constraint of the published trajectory sequence which is defined based on the real trajectory sequence and the autocorrelation noise sequence. It ensures the consistency and indistinguishability of the published trajectory sequence and the original trajectory sequence and the noise sequence, which further ensures privacy security.

**Theorem 1.** If the cross-correlation function of the published trajectory sequence in any two consecutive moments is superposed by the autocorrelation functions of the original trajectory sequence and the noise sequence after adding the latter (i.e., the temporal cross-correlation of the published trajectory sequence is constrained), then:

$$R_{\left(t_{i},t_{i}+1\right)}^{\left(z_{i},z_{i}+1\right)}=R_{\left(t_{i},t_{i}+1\right)}^{X}+R_{\left(t_{i},t_{i}+1\right)}^{Y}$$ (9)

**Proof.** The theorem is provable by mathematical induction. First, the timing cross-correlation of published trajectory sequence is proven feasible at time points t_0_ and t_1_. Then, it is assumed that the timing cross-correlation of published trajectory sequence is feasible at time points t_n-1_ and t_n_. The temporal cross-correlation of the published trajectory sequence is thus feasible at t_n_ and t_n+1_. Thereby, the temporal cross-correlation of the published trajectory sequence which meets the theorem constraints at any time is feasible.

The original trajectory sequence is represented as X(t). The noise sequence be represented as Y(t). After adding the noise sequence, the published trajectory sequence is represented as Z(t), then Z(t) = X(t)+ Y(t). τ is the time interval, τ>0.

(1) Set the time interval τ, where in t_0_ and t_1_, t_1_ = t_0_+τ. The published trajectory sequence is expressed as:

$$Z_{0}=X_{0}(t_{0})+Y_{0}(t_{0})$$ (1-1)

$$Z_{1}=X_{1}(t_{1})+Y_{1}(t_{1})=X_{0}(t_{0}+\tau )+Y_{0}(t_{0}+\tau )$$ (1-2)

Where X_0_(t_0_) and X_1_(t_1_) are the original user trajectories in t_0_ and t_1_, Y_0_(t_0_) and Y_1_(t_1_) are added Laplacian noise elements in t_0_ and t_1_.

The autocorrelation function of the original trajectory sequence is:

$$X_{01}=E[X(t_{0})X(t_{1})]=\frac{1}{\tau }\int_{-\tau }^{\tau }\left(X_{0}(t_{0})X_{0}(t_{0}+\tau \right))dt$$ (1-3)

The autocorrelation function of the noise sequence is:

$$Y_{01}=E[Y(t_{0})Y(t_{1})]=\frac{1}{\tau }\int_{-\tau }^{\tau }\left(Y_{0}(t_{0})Y_{0}(t_{0}+\tau \right))dt$$ (1-4)

At time points t_0_ and t_1_, according to Definition 11, the cross-correlation function of the published trajectory sequence Z is:

$$\begin{array}{l} Z_{01}=\frac{1}{\tau }\int_{-\tau }^{\tau }Z_{0}\cdot Z_{1}dt\\ =\frac{1}{\tau }\int_{-\tau }^{\tau }\left(\left(X_{0}(t_{0})+Y_{0}(t_{0}\right)\right)\cdot (X_{0}(t_{0}+\tau )+Y_{0}(t_{0}+\tau )))dt\\ =\frac{1}{\tau }\int_{-\tau }^{\tau }\left(X_{0}(t_{0})X_{0}(t_{0}+\tau )+X_{0}(t_{0})Y_{0}(t_{0}+\tau )+Y_{0}(t_{0})X_{0}(t_{0}+\tau )+Y_{0}(t_{0})Y_{0}(t_{0}+\tau )\right)dt \end{array}$$ (1-5)

Because t_0_ is the starting time and t_1_ = t_0_+τ, the same function sequence X is the timing cross-correlation at t_0_ and t_0_+τ, the same function sequence Y is also the timing cross-correlation at t_0_ and t_0_+τ. The function sequences X and Y are not temporally cross-correlated at t_0_ and t_0_+τ, that is, X_0_(t_0_) are not related to Y_0_(t_0_+τ), Y_0_(t_0_) are not related to X_0_(t_0_+τ), and their correlation coefficient value is 0.

That is, X_0_(t_0_) Y_0_(t_0_+τ) = 0, Y_0_(t_0_)X_0_(t_0_+τ) = 0.

Therefore, Eq (1-5) can be expressed as:

$$\begin{array}{l} Z_{01}=\frac{1}{\tau }\int_{-\tau }^{\tau }\left(X_{0}(t_{0})X_{0}(t_{0}+\tau )+Y_{0}(t_{0})Y_{0}(t_{0}+\tau )\right)dt\\ =E[X(t_{0})X(t_{1})]+E[Y(t_{0})Y(t_{1})]\\ =X_{01}+Y_{01} \end{array}$$ (1-6)

Z_01_ = X_01_+Y_01_, so the timing cross-correlation of the issuance trajectory sequence is feasible at t_0_ and t_1_.

(2) Assume that the timing cross-correlation of published trajectory sequence is feasible at time points t_n-1_ and t_n_(t_n_ = t_n-1_+τ). According to Definition 11, the cross-correlation function of the published trajectory sequence Z_(n-1)n_ is:

$$\begin{array}{l} Z_{(n-1)n}=\frac{1}{\tau }\int_{-\tau }^{\tau }Z_{n-1}\cdot Z_{n}dt\\ =\frac{1}{\tau }\int_{-\tau }^{\tau }\left(\left(X_{n-1}(t_{n-1})+Y_{n-1}(t_{n-1}\right)\right)\cdot (X_{n-1}(t_{n-1}+\tau )+Y_{n-1}(t_{n-1}+\tau )))dt\\ =\frac{1}{\tau }\int_{-\tau }^{\tau }\left(Y_{n-1}(t_{n-1})X_{n-1}(t_{n-1}+\tau )+Y_{n-1}(t_{n-1})Y_{n-1}(t_{n-1}+\tau )\right)dt\\ +\frac{1}{\tau }\int_{-\tau }^{\tau }\left(Y_{n-1}(t_{n-1})X_{n-1}(t_{n-1}+\tau )+Y_{n-1}(t_{n-1})Y_{n-1}(t_{n-1}+\tau )\right)dt \end{array}$$ (1-7)

The timing cross-correlation of the trajectory sequence is feasible at t_n-1_ and t_n_.

That is, Z_(n-1)n_ = X_(n-1)n_ +Y_(n-1)n_.

Therefore, Eq (1-7) can be expressed as:

$$\begin{array}{l} Z_{(n-1)n}=\frac{1}{\tau }\int_{-\tau }^{\tau }\left(X_{n-1}(t_{n-1})X_{n-1}(t_{n-1}+\tau )+Y_{n-1}(t_{n-1})Y_{n-1}(t_{n-1}+\tau )\right)dt\\ =E[X(t_{n-1})X(t_{n})]+E[Y(t_{n-1})Y(t_{n})]\\ =X_{(n-1)n}+Y_{(n-1)n} \end{array}$$ (1-8)

Therefore, at t_n_ and t_n+1_, there is no autocorrelation between the different functions X_n_(t_n_) and Y_n_(t_n_+τ) or Y_n_(t_n_) and X_n_(t_n_+τ). There is no timing correlation among them and their mutual cross-correlation coefficient is 0. That is, X_n_(t_n_)Y_n_(t_n_+τ) = 0, Y_n_(t_n_) X_n_(t_n_+τ) = 0.

(3) In t_n_ and t_n+1_, t_n+1_ = t_n_+τ, the published trajectory sequence is:

$$Z_{n}=X_{n}(t_{n})+Y_{n}(t_{n})$$ (1-9)

$$Z_{n+1}=X_{n+1}(t_{n+1})+Y_{n+1}(t_{n+1})=X_{n}(t_{n}+\tau )+Y_{n}(t_{n}+\tau )$$ (1-10)

Where X_n_(t_n_) and X_n+1_(t_n+1_) are the original trajectory sequences at t_n_ and t_n+1_, and Y_n_(t_n_) and Y_n+1_(t_n+1_) are the noise sequences which are added at t_n_ and t_n+1_.

The autocorrelation function of the original trajectory sequence is:

$$X_{n(n+1)}=E[X(t_{n})X(t_{n+1})]=\frac{1}{\tau }\int_{-\tau }^{\tau }\left(X_{n}(t_{n})X_{n}(t_{n}+\tau \right)dt$$ (1-11)

The autocorrelation function of the noise sequence is:

$$Y_{n(n+1)}=E[Y(t_{n})Y(t_{n+1})]=\frac{1}{\tau }\int_{-\tau }^{\tau }\left(Y_{n}(t_{n})Y_{n}(t_{n}+\tau \right)dt$$ (1-12)

At time points t_n_ and t_n+1_, according to Definition 11, the cross-correlation function of the published trajectory sequence Z_(n-1)n_ is:

$$\begin{array}{l} Z_{n(n+1)}=\frac{1}{\tau }\int_{-\tau }^{\tau }Z_{n}\cdot Z_{n+1}dt\\ =\frac{1}{\tau }\int_{-\tau }^{\tau }\left(\left(X_{n}(t_{n})+Y_{n}(t_{n}\right)\right)\cdot (X_{n}(t_{n}+\tau )+Y_{n}(t_{n}+\tau )))dt\\ =\frac{1}{\tau }n\int_{-\tau }^{\tau }\left(X_{n}(t_{n})X_{n}(t_{n}+\tau )+X_{n}(t_{n})Y_{n}(t_{n}+\tau )+Y_{n}(t_{n})X_{n}(t_{n}+\tau )+Y_{n}(t_{n})Y_{n}(t_{n}+\tau )\right)dt \end{array}$$ (1-13)

Because the temporal cross-correlation of the published trajectory sequence is feasible at t_n-1_ and t_n_, there is no autocorrelation between the functions X_n_(t_n_) and Y_n_(t_n_+τ) or Y_n_(t_n_) and X_n_(t_n_+τ), and there is no temporal correlation among them.

Their mutual cross-correlation coefficient is 0, i.e., X_n_(t_n_)Y_n_(t_n_+τ) = 0, Y_n_(t_n_) X_n_(t_n_+τ) = 0.

Therefore, Eq (1-13) can be expressed as:

$$\begin{array}{l} Z_{n(n+1)}=\frac{1}{\tau }\int_{-\tau }^{\tau }\left(X_{n}(t_{n})X_{n}(t_{n}+\tau )+Y_{n}(t_{n})Y_{n}(t_{n}+\tau )\right)dt\\ =E[X(t_{n})X(t_{n+1})]+E[Y(t_{n})Y(t_{n+1})]\\ =X_{n(n+1)}+Y_{n(n+1)} \end{array}$$ (1-14)

Therefore, the temporal cross-correlation of the published trajectory sequence Z_n(n+1)_ is feasible at t_n_ and t_n+1_.

In summary, the temporal cross-correlation of the published trajectory sequence is feasible and meets the theorem constraints as-established at any time. The cross-correlation function $R_{{(t}_{i}{,t}_{i+1})}^{{(z}_{i}{,z}_{i+1})}$ of the published trajectory sequence in any two consecutive time points t_i_ and t_i+1_, which is the superposition of the autocorrelation function of the original trajectory sequence $R_{{(t}_{i}{,t}_{i+1})}^{X}$ and the noise sequence $R_{{(t}_{i}{,t}_{i+1})}^{Y}$ in these two moments, indicates that the published trajectory sequence is temporally cross-correlated and indistinguishable.

### 4.3 Privacy protection method based on cross-correlation constraint

If the published trajectory sequence meets the cross-correlation constraint at any two consecutive moments, then the consistency of the published trajectory sequence with the original trajectory and noise sequences can be guaranteed in logical and spatio-temporal domains, which makes them indistinguishable. As a result, the attacker cannot identify the real user trajectory from the published trajectory sequence. This achieves the "absolute" security of private data.

According to Theorem 1, when the published trajectory sequence meets the cross-correlation constraint in any two consecutive moments, it is required that the published trajectory sequence satisfies the cross-correlation constraint while the original trajectory sequence and noise sequence satisfy the autocorrelation constraint. The cross-correlation published trajectory sequence is superposed by the autocorrelation function of the original trajectory sequence and the noise sequence at these two moments.

Here, four groups of Gaussian white noise Y_1_、Y_2_、Y_3_、Y_4_(λ = 1,2,3,4) are transformed into a group Laplace noise sequence by Laplacian transformation of Lap(2λ^2^) [20]. When the autocorrelation function R_Y_'(τ) of the noise sequence and the autocorrelation function R_XX_ (τ) of the original trajectory sequence satisfy the condition R_XX_ (τ) = 8 R_Y_'(τ)^2^, the added noise and original trajectory meet the timing correlation [1]. The Laplacian noise sequence which satisfies the autocorrelation requirement in this study is generated accordingly. Then, according to the cross-correlation constraint mechanism, the noise sequence is superimposed into the user's real trajectory sequence to form a published trajectory sequence which meets the cross-correlation constraint for the purposes of privacy protection.

In practice, the user's trajectory sequence is processed into a short-term stable sequence according to a time interval with a certain frequency. Filtering and signal processing methods are then deployed to add noise and disturbance. This ultimately forms a published trajectory sequence which meets the cross-correlation constraints (Algorithm 2).

**Algorithm 2**. Generate a published trajectory sequence

**Input**: original trajectory sequence X, privacy budget Ɛ_i_, Time interval T

**Output**: published trajectory sequence Z

1 ∀ ℇ_i_∈ℇ

2 ∃ Y_j_∈Y (j = 1,2,3,4) Generate Gaussian white noise according to Y_j_'~N(0, √2λ^2^)

$$L(D)=f(D)+Laplace(\frac{\Delta f}{\varepsilon_{i}})\;\mathrm{Pr}[x]=\frac{1}{2\lambda }\exp^{\left(-\frac{\left|x-\mu \right|}{\lambda }\right)}$$

3 Calculate the autocorrelation function R_XX_(τ) of the user's real trajectory sequence X

4 Calculate the autocorrelation function of the addednoise sequence Y $R_{Y}\left(\tau \right)=\sqrt{\frac{R_{XX}\left(\tau \right)}{8}}$

5 Based on the autocorrelation function Ry(τ) of the noise sequence Y, Y_j_' is converted into a new correlation Gaussian white noise Y_j_ (j = 1,2,3,4), Y_j_~N(0,2λ^2^) /* Filter is $h\left(\tau \right)=\sqrt{\frac{R_{XX}\left(\tau \right)}{16\pi N}}$

6 ${Y=Y}_{1}^{2}{+Y}_{2}^{2}{‐Y}_{3}^{2}{‐Y}_{4}^{2}$ /*Synthesize the Laplacian noise sequence according to the timing correlation

7 Z = X+Y /* According to the constraint condition $R_{{(t}_{i}{,t}_{i+1})}^{{(z}_{i}{,z}_{i+1})}=R_{{(t}_{i}{,t}_{i+1})}^{X}+R_{{(t}_{i}{,t}_{i+1})}^{Y}$, generating a trajectory sequence Z.

8 Return the published trajectory sequence Z.

### 4.4 Analysis of privacy protection method

The cross-correlation constraints ensure the consistency of the published trajectory sequence with the original trajectory and noise sequences in logical and spatio-temporal domains. This makes them indistinguishable, the attacker cannot identify the actual user trajectory information from the published trajectory sequence.

In this paper, a (R, Ɛ)-differential privacy protection model is established based on the Ɛ-differential privacy protection method. By protecting sensitive location points and affiliated sensitive points on the user's trajectory, the sensitivity of location points is combined with user requirements and privacy budgets. The user's location and privacy requirements are utilized to add differential Laplacian noise, then differential privacy parameters are set accordingly. By introducing the privacy model parameter R, the differential privacy budget parameter Ɛ can be changed corresponding to the privacy protection level of the given location points. It allows noise of varying intensity to be dynamically added for personalized, differential privacy protection.

A spatio-temporal correlation and varying-intensity noise sequences are added to the user's real trajectory sequence under the cross-correlation constraint condition. The real trajectory sequence and noise sequence which satisfy the autocorrelation are superimposed to form a published trajectory sequence, which ensures consistency between the published trajectory sequence with the original trajectory and noise sequences in logical and spatio-temporal domains.

The proposed method efficiently protects the user's private information while making the published trajectory data highly available in the following five steps.

#### Step 1: Construct a trajectory semantic map

The entire city map is meshed into a semantic map which is then divided into several distinct areas. The areas are numbered to form the semantic map topology of the mobile user.

#### Step 2: Trajectory data preprocessing

The trajectory semantic map and initial parameters are used to divide the location points into {sensitive location set, non-sensitive location set, logical location set} in the map area. An undirected graph of the trajectory area is then constructed accordingly. The privacy level value of each vertex in the trajectory graph is set, then the weight of the adjacent edge and the privacy level value of the affiliated sensitive point are calculated.

#### Step 3: Calculate the location point corresponding to the privacy budget Ɛ_i_

According to the (R,Ɛ)-differential privacy protection model condition, the privacy budget Ɛ_i_ corresponding to each location point is calculated.

#### Step 4: Anonymize the trajectory data

According to the defined time interval and privacy budget, the differential privacy protection method is used to add Laplacian noise with correlation and varying scrambling intensity to each location point to be protected. This generates a published trajectory sequence based on cross-correlation constraints $R_{{(t}_{i}{,t}_{i+1})}^{{(z}_{i}{,z}_{i+1})}=R_{{(t}_{i}{,t}_{i+1})}^{X}+R_{{(t}_{i}{,t}_{i+1})}^{Y}$.

#### Step 5: Publish trajectory data

The obtained cross-correlation trajectory sequence is published by third parties.

## 5 Experimental evaluation and results analysis

The proposed method was evaluated experimentally in terms of privacy security, data availability and execution efficiency. The proposed method (referred to from here on as the “DP-UR”) was compared against a method proposed by Wang Hao [1] (the “CLM”) and another by Chen Rui [21] (the “P-refix”). The DP-UR, CLM and P-refix all involve adding noise to the real trajectory to protect private information.

### 5.1 Experimental design and parameter settings

In order to evaluate the feasibility and effectiveness of the proposed method, it verifies the three aspects of privacy security, data availability and execution efficiency. It compares the proposed method with other methods under the same experimental parameters to further determine the efficiency. The design of the experimental process is divided into horizontal and vertical levels. At the horizontal level, it sets the same environment and parameters, and it compares the proposed method with the other methods to evaluate its performance and efficiency. At the vertical level, by setting different parameters and environments, it tests the performance of the proposed method and evaluates the best performance.

**Fig 5:**
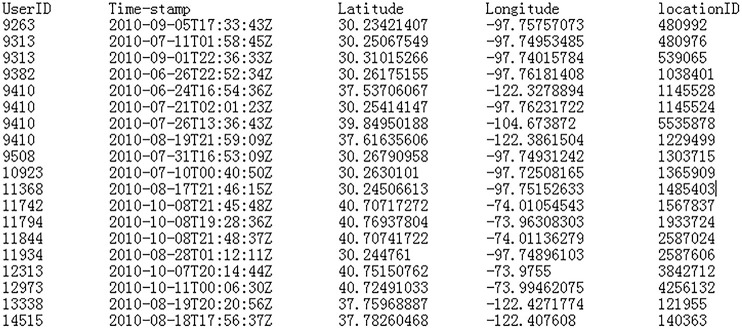
Data set. .

In this paper, the experiment is conducted on a computer with an Intel Core (TM) i5-3470 CPU @ 3.2 GHz and 8 GB RAM running over the Microsoft Windows 7.sp1.64 bit operating system. The algorithm is implemented in Visual C + + 6.0. The user's check-in records in Gowalla dataset from February 2009 to October 2010 is used as experimental data sets to perform the corresponding experimental verification. It contains 950,327 check-in records for 6,442,890 users in One and a half years [22], the data set is shown in Fig 5.

Where 1st column represents the user's identification ID, the 2nd column represents the time when the user is at the current position, the 3rd and 4th columns represent the spatial coordinates of the user's current location, and the 5th column represents the location region number corresponding to the location point. The used parameters in the experiment are shown in Table 1.

**Table 1:** Parameters of experiment.

Variable Name	Description	Default Values	Step
ε	privacy budge	[0.1, 1]	0.1
R	privacy model parameter	[0.1, 1]	0.1
m	sensitive location set	[10,40]	5
pv	privacy level value	[0,1]	0.1

### 5.2 Experimental results and analysis

#### 5.2.1 Security analysis

Security in this context mainly refers to the probability that the user's real trajectory data is recognized by an attacker. The actual differential privacy budget directly reflects the privacy protection level. A smaller Ɛ value indicates that more noise has been added. The published trajectory data is more secure when the level of privacy protection is higher.

**Fig 6:**
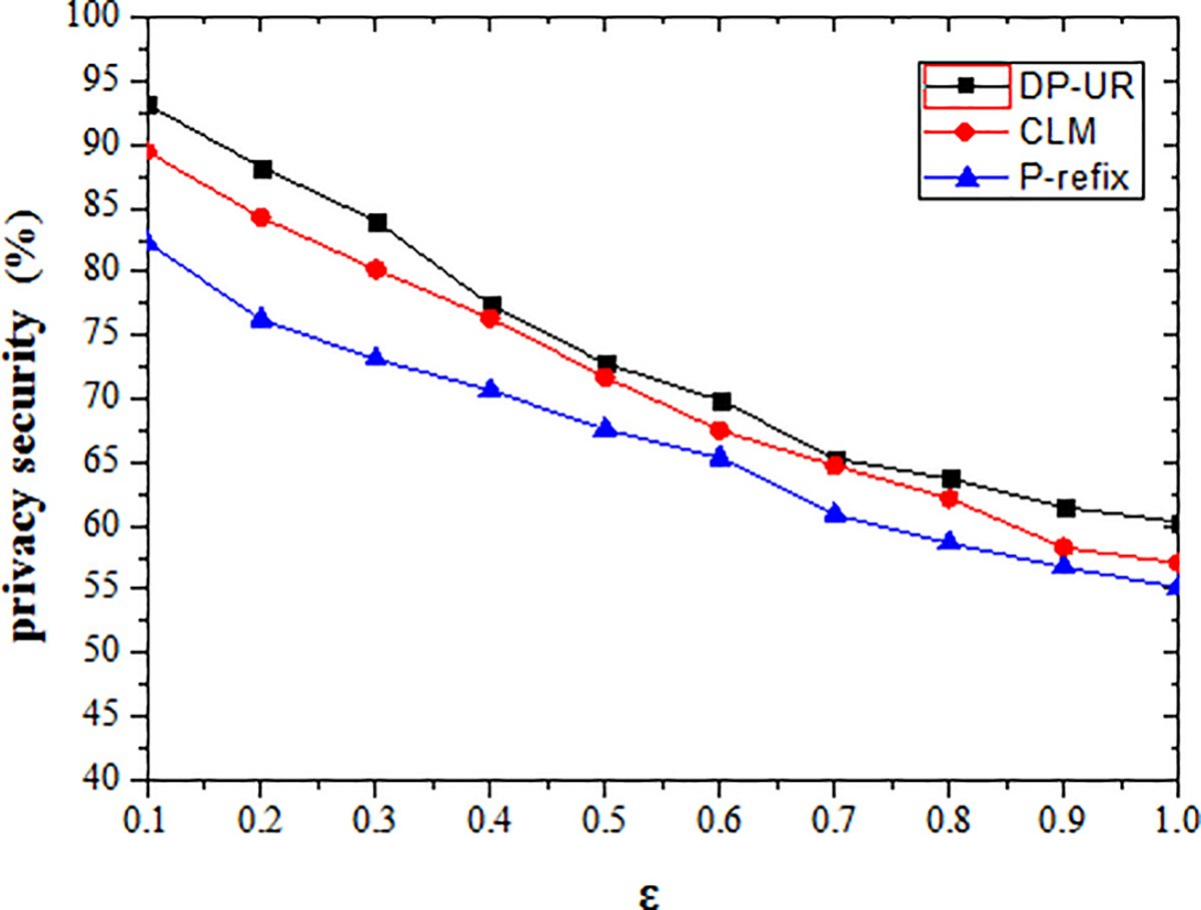
Effect of privacy budget parameter on security.

The disturbance of the original trajectory data is diminished by the differential privacy method as the privacy protection budget value increases, while the level of privacy protection is reduced, as shown in Fig 6. The P-refix method achieves the lowest degree of privacy protection, as it randomly adds noise through the privacy budget (and the privacy protection effect is closely related to the amount of noise). The added noise is independently and equally distributed in low correlation with the original trajectory data. The attacker can reduce the disturbance caused by noise by filtering, so the privacy protection effect is relatively low. When the disturbed noise is added, the CLM and DP-UR methods consider the temporal correlation between the added noise sequence and the original trajectory sequence. They make the original trajectory sequence, added noise sequence and published trajectory sequence unrecognizable to a certain extent for better privacy protection than P-refix.

Privacy protection is inversely proportional to the privacy budget Ɛ, the smaller Ɛ means larger data distortion, and the higher the degree of privacy protection. When Ɛ tends to zero, the privacy protection reaches the highest level in theory. As can be seen from the experimental results, when Ɛ = 0.1, the privacy protection of the proposed method can reach 93.19%, and the other two methods are respectively 89.46% and 82.34%. When Ɛ = 1, the privacy protection of the proposed method is 60.29%, and the other two methods are respectively 57.06% and 55.16%. Overall, the privacy protection of proposed method has improved by an average of 2.45% and 6.96%.

The DP-UR comprehensively considers the temporal correlation between the original trajectory sequence and the noise sequence. It ensures the logical consistency of the added noise sequence and original trajectory sequence while properly accounting for cross-correlation of the published trajectory sequence itself. As per the cross-correlation constraint, it guarantees the consistency of the published trajectory sequence with the original trajectory and noise sequences in the spatio-temporal domain. It provides effective privacy protection and makes the user's real, private trajectory information more secure. The DP-UR sets different privacy budget values Ɛ_i_ according to the varying sensitivity of the location points in the trajectory. It allows for dynamic adjustments based on the user's specific requirements, which provides stronger privacy protection than the other two methods tested in this study.

For a given privacy model parameter R, the larger the privacy level value, the smaller the privacy budget, and the privacy protection is higher. When the user is located in a particularly sensitive area, the PV tends to 1, the value of the privacy model parameter R is the same as Ɛ, and the privacy protection is inversely proportional to R. It can be seen from the experimental results, when R = 0.1, the privacy protection is 86.02%. When R = 1, the privacy protection is 64.18%.

**Fig 7:**
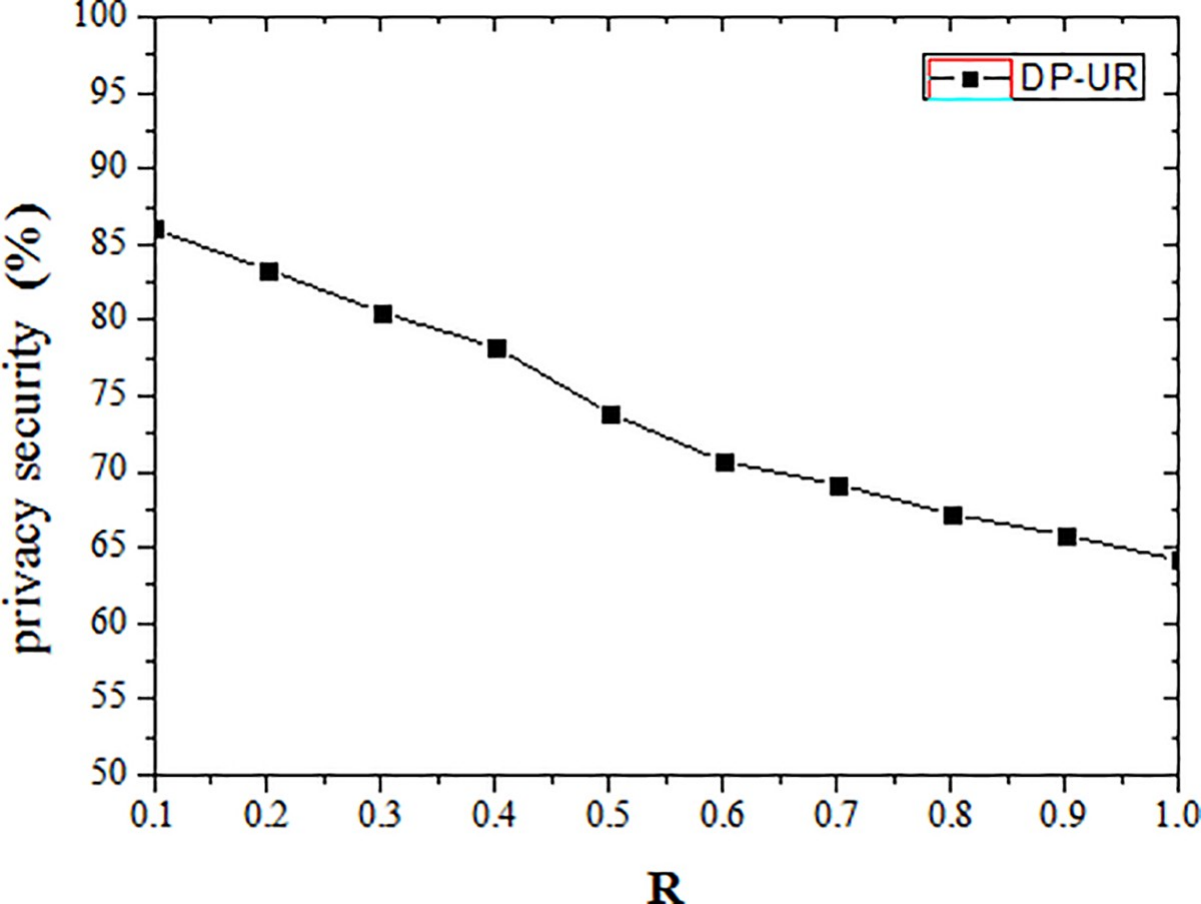
Effect of privacy model parameter on security.

The implemented privacy protection effect decreases as the privacy model parameter R increases, as shown in Fig 7. The DP-UR assigns a certain privacy level value according to the sensitivity of location points on the user trajectory, so the privacy level of the same user at the same location is fixed. A larger privacy model parameter value produces a larger corresponding privacy budget value under a fixed privacy level. Thus the added noise is reduced, as is the privacy protection effect.

It can be seen from experiments and safety analysis, the DP-UR method is effective in differential privacy protection, the achieved degree of privacy protection achieved has increased by an average of 4.71% over the comparative experiment method. It can be seen that the cross-correlation of published trajectory data has a great impact on privacy protection.

#### 5.2.2 Data availability

Data availability is generally measured by information loss of published trajectory data. In this study, information loss is measured by the standard deviation of the distance error between a location point on the user's real trajectory and published trajectory. The Manhattan distance calculation method is very consistent with the actual environment of mobile users [23], so it is used here. The usability of the published trajectory data is lower when more information is lost from the published trajectory:

$$\mathrm{DU}=\sqrt{\frac{\sum_{i=1}^{n}{\left(d_{i}-\bar{d}\right)}^{2}}{\left|L\right|}}$$ (10)

where *d*_*i*_ is the Manhattan distance between the position *loc*_*i*_ on the real trajectory and the corresponding position point *loc* on the published trajectory, $\bar{d}$ is the average Manhattan distance between them, *n* is the number of position points on the trajectory, and *| L|* is the size of the trajectory.

Ɛ is a key parameter in differential privacy, it is used to determine the strength of privacy protection and the amount of added noise. According to Dwork's research, when Ɛ tends to 1, data availability could reach a more appropriate level. In the experiment, the availability of data is tested with different Ɛ, when the value range of Ɛ is [0.1, 1] and the step size is 0.1. It can be seen from the experiment, as Ɛ is increased, the availability of data is improved. When Ɛ = 0.1, the data availability of the proposed method is 65.31%, it is on average 4.27% higher than the other two methods. When Ɛ = 1, the data availability is 90.09%, it is on average 7.36 higher than the other two methods.

**Fig 8:**
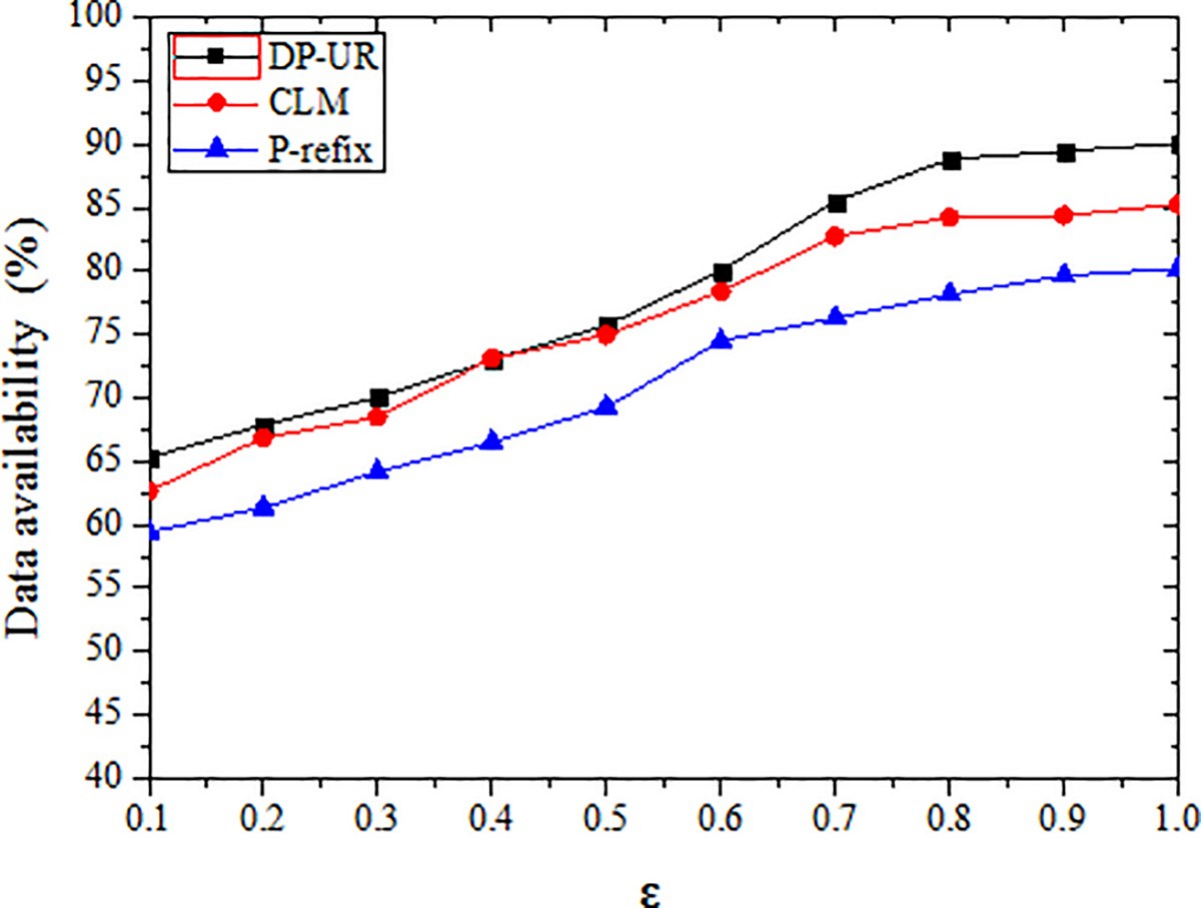
Effect of privacy budget parameter on data availability.

The data availability of all three methods increased as the privacy protection budget value increased in this experiment, as shown in Fig 8. Because the amount of added noise in the published trajectory is also reduced as Ɛ increases, the amount of information lost due to privacy protection is gradually reduced, the query result grows more accurate, and the availability of published data increases.

When the initial privacy budget is the same, the DP-UR appears to provide higher data availability than the other two methods tested in this study. Because noise is randomly (and uncontrollably) added in the P-refix method, the generated information loss is large. The CLM method adds the same intensity of noise to all location points, so the same intensity is disturbed for all trajectories and information loss is relatively large. The DP-UR method adds appropriate and differential Laplacian noise according to the sensitivity of location points in the user’s trajectory. Differential noise disturbance also depends on the sensitivity of the trajectory. Thus, the information loss is relatively small and the availability of the trajectory data is relatively high.

According to the constraint pv×Ɛ = R in (R, Ɛ)-differential privacy protection model, when pv is constant, R is proportional to Ɛ. With the smaller R, the Ɛ value is also smaller, it means more noise, which corresponds to more privacy and less utility, whilst larger R means the opposite. It can be seen from the experiment, when R = 0.1, the data availability is 75.16%, when R = 1, the data availability is 89.59%.

**Fig 9:**
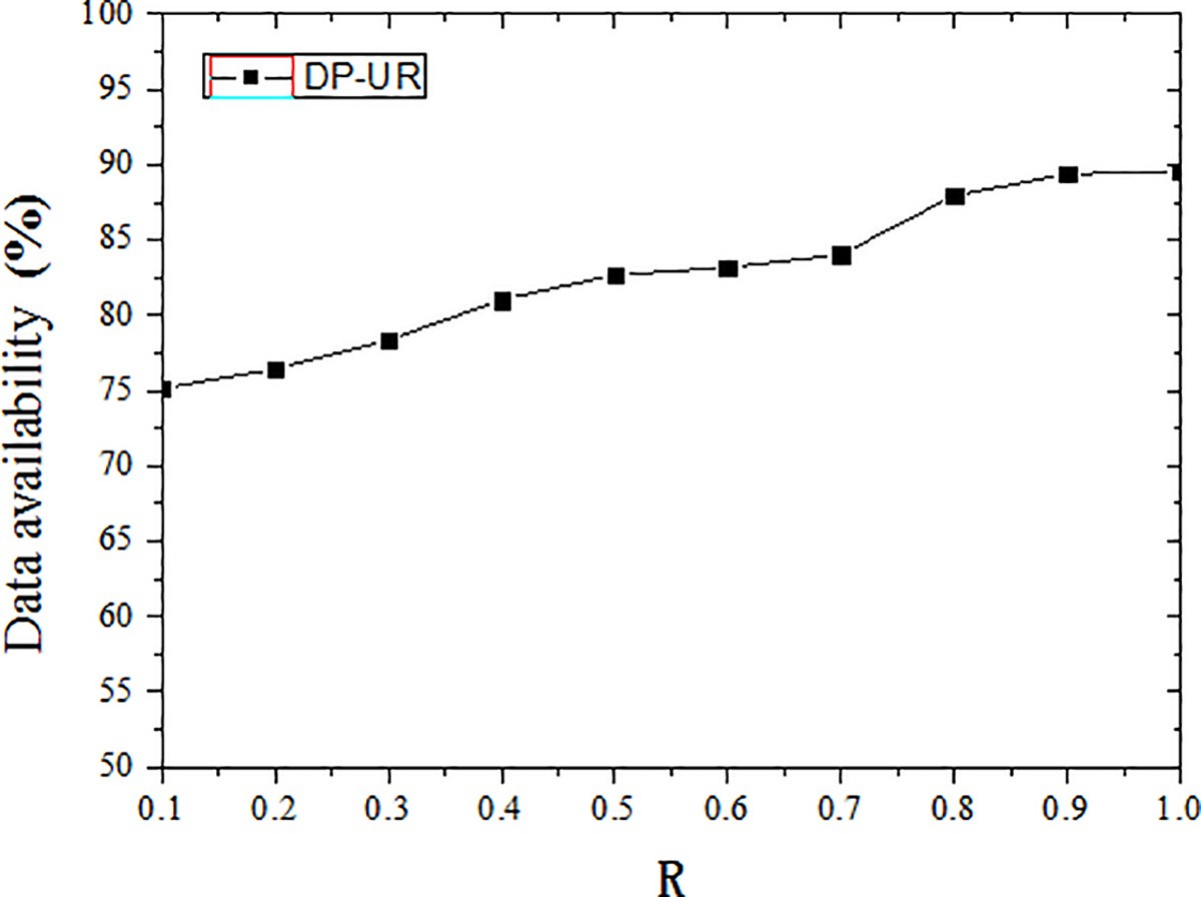
Effect of privacy model parameter on data availability.

The availability of the published trajectory data increases as privacy model parameter R increases, as shown in Fig 9. The corresponding differential privacy budget value increases as R increases as well, which drives down the added disturbance noise. In this case, the degree of disturbance to the original trajectory data is reduced, the information loss is reduced, and the availability of data is improved.

It can be seen from experiments and data availability analysis, the proposed DP-UR method can achieve good data availability. When the degree of privacy protection increases, the data availability of DP-UR method is significantly higher than the comparative experiment, it has increased by an average of 5.06%. It is possible to set an appropriate level of privacy protection, and ensure the good data availability, to achieve the privacy-utility trade-off.

#### 5.2.3 Execution efficiency

**Fig 10:**
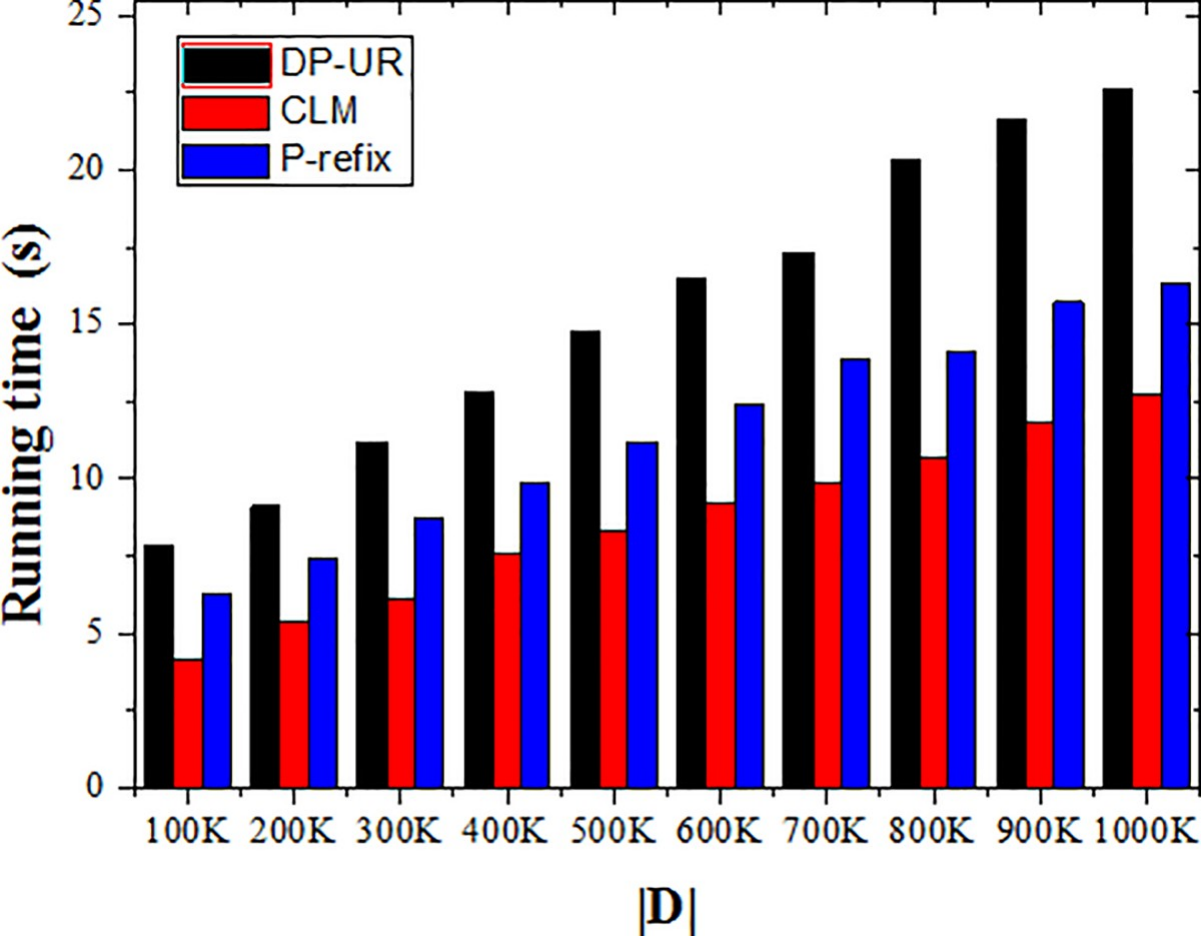
Effect of data set size on execution efficiency.

In this paper, execution efficiency mainly refers to the time required for privacy protection. As shown in Fig 10, the execution time of a given privacy protection method is related to the size of the trajectory data set. In this experiment, it increased approximately linearly as the data set increased; this was expected as a larger data set requires longer processing time. As shown in Fig 10, the execution time of the proposed method is slightly longer than the other two methods.

The DP-UR method protects the sensitive location points of the trajectory, so it also protects the affiliated sensitive points and sets different budget parameters ℇ according to the sensitivity level while varying-intensity Laplacian noise is added for disturbance. In the process of adding disturbing noise, the temporal correlation between the added noise sequence and the original trajectory sequence is considered in addition to the cross-correlation of the published trajectory sequence itself. This ensures consistency between the published trajectory sequence and the original trajectory and noise sequences in the spatio-temporal domain. There are more constraints in the process of trajectory privacy protection in this case, so the execution time is relatively lengthy.

The execution time is closely related to the time complexity and calculation complexity of the algorithm. Under the same data scale, the calculation complexity of the proposed DP-UR method is larger than the other two methods, so the execution time is also longer than them. It can be seen from the experiment that the average increase is 42.63% in execution time.

#### 5.2.4 Experimental results

The experiments discussed above altogether indicated that the proposed method can provide efficient privacy protection for user trajectories and ensure high availability for published trajectory data. Different privacy level values are assigned as necessary and different noise parameters are set according to the sensitivity of certain position points and the affiliated sensitive points of the trajectory. Various intensity disturbances are utilized to reduce information loss from the published trajectory data and to ensure high data utilization.

When disturbing noise is added, the proposed method not only defines the temporal correlation between the added noise sequence and the original trajectory sequence but also defines the cross-correlation constraint of the published trajectory sequence. This results in strong privacy protection effects. Several factors in the anonymity process are taken into account, so the execution efficiency is relatively low. However, with the continuous improvement of hardware devices, this method can be expected to meet reasonable user requirements.

Furthermore, the proposed method is compared with previous and current literature in the same field on basis of method, merits, weakness and Ɛ allocation is presented in Table 2.

**Table 2:** Comparison of privacy preservation strategies.

Privacy Name	Year	Privacy Mechanism	Merits or Advantages	Weakness and Challenges	Noise mechanism Ɛ allocation
DP-UR (proposed method)	/	Cross-correlation Constraint	Protecting sensitive and affiliated points, adding differential noise based on location sensitivity, ensuring the consistency of published trajectory sequence.	Algorithm's time complexity is high.	Laplacian mechanism.Ɛ_i_ = R / PV_i_
Laplace [4]	2006	Query processing based on differential privacy	It supports range query per unit length, the calculation time is small, and it is only used to add noise.	The published deviation is large, the usability of the histogram is low, and the actual accuracy is poor.	Laplace mechanism. Ɛ is uniformly distributed.
α-DP_T_ [24]	2017	Markov modeling method	Considering the time correlation of the data, the time polynomial is used to calculate the privacy loss.	When the length of the release time is unknown, the α-DP_T_ method will be difficult to implement.	Laplacian mechanism. Ɛ is evenly distributed.
Kanonymity [25]	2020	K-anonymity	The operation is simple, the calculation overhead is small, and the algorithm is highly portable.	Low privacy guarantee, dependent on the background knowledge of attackers.	/
DQAM [26]	2019	Trajectory histogram partition	Adaptively add noise, high query accuracy, low deviation rate.	Only applicable to spatial histograms, not applicable to any spatial distribution.	Laplace or exponential mechanism. Ɛ is split according to the stages.
DPLRM [27]	2018	Markov probability method	Using Markov chain to model. Considered the timing correlation of the position points on the trajectory.	The time complexity is high, the processing time is long, and the real-time performance is poor.	Laplace or exponential mechanism. Ɛ is dynamically allocated.
Noisy-QT Cons-QT [28]	2018	Quad-tree R-tree	The trajectory position data at any time meets the requirements of differential privacy.	The time complexity is high, and the generated deviation on small-scale data sets is large.	Laplace mechanism. Ɛ is divided according to the level of tree.
Kalman [29]	2018	Time series data release based on filtering technology	Using Kalman technique to filter and add noise, the availability of data is good.	It is difficult to determine the sampling interval.	Laplace mechanism. Ɛ is randomly assigned.
DP-topkP [30]	2014	Consistency constraints of differential privacy	The output results meet the consistency constraints and the data accuracy is high.	The performance and efficiency are relatively poor when dealing with large values of k or l.	Laplace or exponential mechanism.Ɛ is evenly distributed.
MB-CI [31]	2017	Weight information privacy protection	The amount of global noise required is less, and the availability of data is good.	The merge operation may reveal privacy.	Laplace mechanism.Ɛ is randomly assigned.
GDD-PD [32]	2016	Social network degree information release	The projection method is used to reduce data sensitivity.	It is not applicable to use low-degree information to predict high-degree nodes.	Laplace mechanism.Ɛ is evenly distributed.
NoiseGraph [33]	2017	The release of composite images	Under a lower privacy budget, the triangle count of the composite graph can still be guaranteed to be accurate.	The evaluation of the k-triangle count is insufficient.	Laplace mechanism.Ɛ is uniformly distributed.

## 6 Conclusion

A personalized differential privacy protection method is proposed, which is to address the issue of adding independent and uncorrelated noise and the same degree of scrambling results in low privacy protection and poor data availability. By combining sensitive location points and their associated sensitive points on the trajectory, as well as the sensitivity of the location on the user trajectory, user privacy protection requirements and privacy protection budget, a (R, Ɛ)-differential privacy protection model is proposed to perform differential anonymization and achieve high efficiency. The constraint mechanism for publishing the cross-correlation of the trajectory sequence is defined, by superimposing the real trajectory sequence on the user’s noise sequence that satisfies the autocorrelation, a published trajectory sequence that satisfies the cross-correlation constraint condition is established. Finally, the feasibility and effectiveness of the algorithm are verified by simulation experiments, and the proposed method is compared with recent studies in the same field on basis of merits and weakness and so on.

However, differential privacy is effective to protect the offline data, but there are certain limitations in protecting online data and real-time data. As the number of queries increases, the allocated privacy budget is reduced, the added noise in the returned data set is too large, which leads to reduced data availability. Furthermore, the balance of privacy-utility trade-off is not dependent solely on differential privacy approach itself, but rather the particular algorithm or used mechanism. Therefore, the research on the differential privacy protection method that can process online data quickly and efficiently, and how to scientifically set acceptable privacy parameters to ensure the availability and consistency of published data which are the contents of the next research.
